# Supplementary figures and images for: Out of (the) bag—encoding categorical predictors impacts out-of-bag samples
Source: PeerJ Comput Sci. 2024 Nov 18;10:e2445. doi: 10.7717/peerj-cs.2445 (PMC11623134; doi:10.7717/peerj-cs.2445)

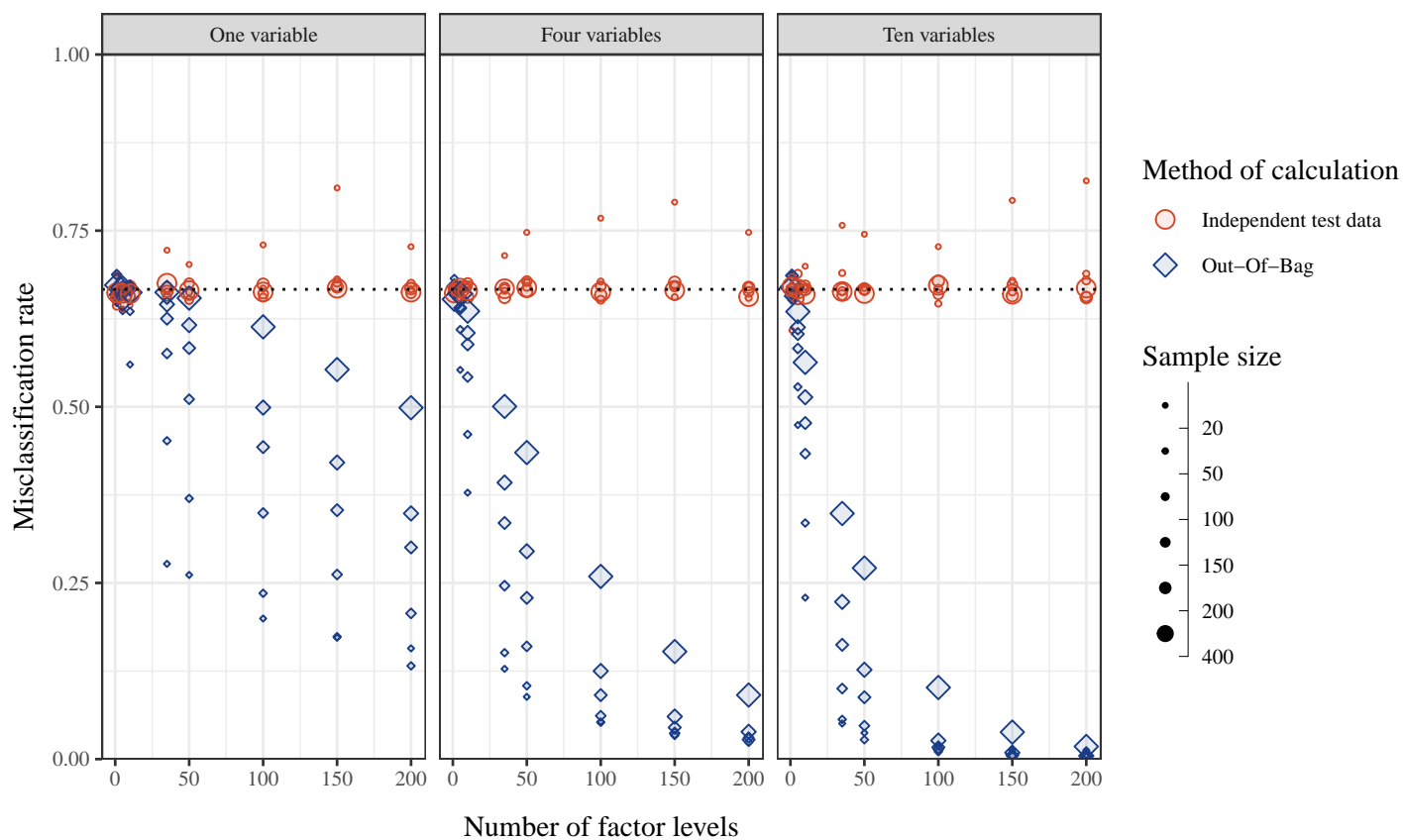

Supplement: Supplemental Information 1 — The effect of method of encoding and increasing number of variables on the out-of-bag misclassification rate. Circles represent misclassification rates calculated using independent test data and diamonds represent misclassification rates calculated using out-of-bag samples. [file peerj-cs-10-2445-s001.pdf]

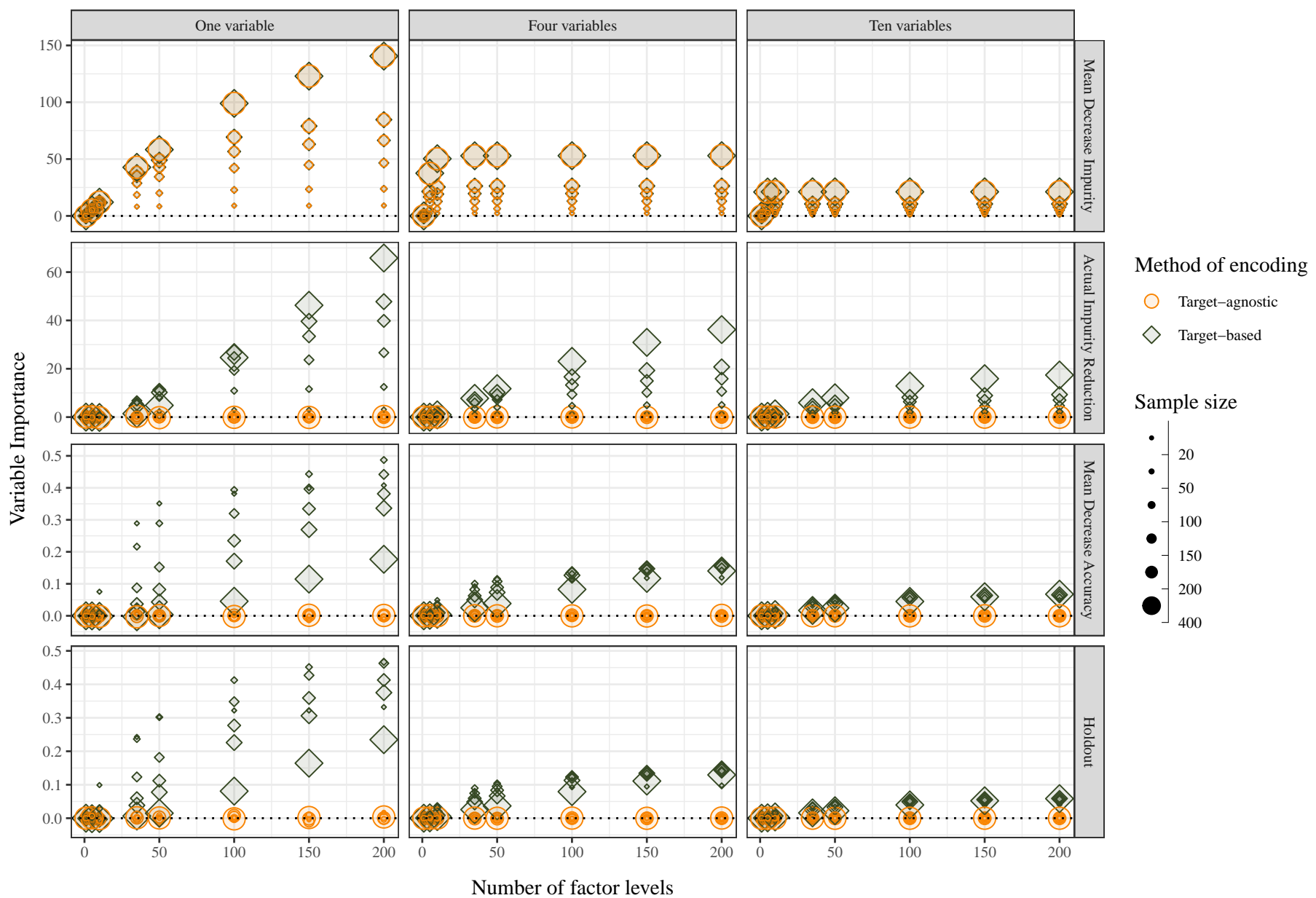

Supplement: Supplemental Information 2 — The effect of method of encoding and increasing number of variables on measures of variable importance. Circles represent variable importance calculated when variables were encoded using a target-agnostic method and diamonds represent variable importance calculated when variables were encoded using a target-based method. [file peerj-cs-10-2445-s002.pdf]
